# Bridging Neurobiology and Artificial Intelligence: A Narrative Review of Reviews on Advances in Cochlear and Auditory Neuroprostheses for Hearing Restoration

**DOI:** 10.3390/biology14091309

**Published:** 2025-09-22

**Authors:** Daniele Giansanti

**Affiliations:** Istituto Superiore di Sanità, Via Regina Elena 299, 00161 Rome, Italy; gianslele2@hotmail.com or daniele.giansanti@iss.it

**Keywords:** artificial intelligence, auditory neuroprosthetics, neural plasticity, cochlear implants, biomarker standardization, auditory neuroscience, biological data integration

## Abstract

Background: Hearing loss results from diverse biological insults along the auditory pathway, including sensory hair cell death, neural degeneration, and central auditory processing deficits. Implantable auditory neuroprostheses, such as cochlear and brainstem implants, aim to restore hearing by directly stimulating neural structures. Advances in neurobiology and device technology underpin the development of more sophisticated implants tailored to the biological complexity of auditory dysfunction. Aim: This narrative review of reviews aims to map the integration of artificial intelligence (AI) in auditory neuroprosthetics, analyzing recent research trends, key thematic areas, and the opportunities and challenges of AI-enhanced devices. By synthesizing biological and computational perspectives, it seeks to guide future interdisciplinary efforts toward more adaptive and biologically informed hearing restoration solutions. Methods: This narrative review analyzed recent literature reviews from PubMed and Scopus (last 5 years), focusing on AI integration with auditory neuroprosthetics and related biological processes. Emphasis was placed on studies linking AI innovations to neural plasticity and device–nerve interactions, excluding purely computational works. The ANDJ (a standard narrative review checklist) checklist guided a transparent, rigorous narrative approach suited to this interdisciplinary, rapidly evolving field. Results and discussion: Eighteen recent review articles were analyzed, highlighting significant advancements in the integration of artificial intelligence with auditory neuroprosthetics, particularly cochlear implants. Established areas include predictive modeling, biologically inspired signal processing, and AI-assisted surgical planning, while emerging fields such as multisensory augmentation and remote care remain underexplored. Key limitations involve fragmented biological datasets, lack of standardized biomarkers, and regulatory challenges related to algorithm transparency and clinical application. This review emphasizes the urgent need for AI frameworks that deeply integrate biological and clinical insights, expanding focus beyond cochlear implants to other neuroprosthetic devices. To complement this overview, a targeted analysis of recent cutting-edge studies was also conducted, starting from the emerging gaps to capture the latest technological and biological innovations shaping the field. These findings guide future research toward more biologically meaningful, ethical, and clinically impactful solutions. Conclusions: This narrative review highlights progress in integrating AI with auditory neuroprosthetics, emphasizing the importance of biological foundations and interdisciplinary approaches. It also recognizes ongoing challenges such as data limitations and the need for clear ethical frameworks. Collaboration across fields is vital to foster innovation and improve patient care.

## Simple Summary

Hearing loss involves complex biological damage to sensory and neural components of the auditory system. Auditory neuroprosthetics aim to restore function by directly interfacing with these neural pathways. Recent advances in artificial intelligence (AI) enhance device performance through biologically informed predictive modeling, signal processing, and surgical planning. However, challenges such as fragmented biological data, lack of standardized biomarkers, and ethical issues limit progress. This review underscores the importance of integrating biological understanding with AI to develop more adaptive, personalized neuroprosthetic interventions that respect the complexity of auditory biology.

## 1. Introduction

### 1.1. Biological Substrates of Hearing Loss and Neuroprosthetic Solutions

Hearing loss can arise from diverse biological insults [1,2,3], affecting multiple levels of the auditory system, from peripheral sensory structures to central neural circuits [2,3]. Identifying the exact sites and mechanisms of damage is essential. It allows us not only to unravel the intricate neurobiology of auditory dysfunction [1,4] but also to inform the design of targeted therapeutic strategies [5].

Cochlear damage is the most common cause of hearing impairment [1,2]. It primarily targets the inner and outer hair cells of the organ of Corti, the cochlea’s sensory epithelium [2,3]. These specialized cells transduce mechanical sound vibrations into electrical neural signals [2]. Exposure to loud noise, aging (presbycusis), infections, ototoxic drugs, and genetic mutations can all lead to hair cell death or dysfunction [1,3]. The result is a compromised auditory transduction process [1,2,3].

Beyond hair cells, degeneration of spiral ganglion neurons—which convey signals from the cochlea to the brainstem via the auditory nerve—further reduces neural transmission [4]. This occurs especially under chronic or neurodegenerative conditions [4,5]. Damage to the cochlear nerve itself, whether caused by trauma, acoustic neuromas, or congenital malformations, interrupts the auditory signal flow to the brain and may require alternative therapeutic approaches [5].

More centrally, lesions of brainstem auditory nuclei and associated pathways—resulting from vascular events, trauma, demyelinating disease, or tumors—can produce central auditory deficits [6]. These are characterized not by a pure sensory loss but by impaired sound processing [6,7]. Finally, cortical damage involving the primary auditory cortex or higher-order associative regions may compromise conscious sound perception, phoneme discrimination, and language comprehension [7,8]. Crucially, such deficits can occur even when peripheral auditory input remains intact [7,8].

The understanding of these biological substrates informs the development of neuroprosthetic devices [9,10]. Cochlear implants (CIs) are the most widely adopted and clinically validated auditory neuroprostheses [9]. They convert acoustic signals into electrical impulses delivered through an electrode array inserted into the cochlea, directly activating spiral ganglion neurons [9].

When the auditory nerve is absent or non-functional, auditory brainstem implants (ABIs) bypass both the cochlea and the auditory nerve, delivering electrical stimulation directly to the cochlear nucleus within the brainstem [10]. Hybrid cochlear implants integrate residual natural hearing with electrical stimulation, optimizing the use of preserved structures while compensating for impaired regions [11].

Next-generation neuroprostheses explore advanced strategies such as high-density electrode arrays, optogenetic interfaces, and cortical implants [12]. Artificial Intelligence-driven (AI-driven) signal processing allows for individualized adaptation, enhancing frequency resolution, speech perception in noise, and overall biocompatibility [13,14].

### 1.2. Artificial Intelligence Meets Auditory Biology: Toward Smarter Neuroprosthetic Hearing—Rationale

The convergence of auditory neuroscience and artificial intelligence (AI) is opening new horizons for neuroprosthetics [13,14]. Modern cochlear implants and related devices are now equipped with digital processing capabilities that go far beyond traditional amplification [13,14]. As AI technologies mature, they hold the potential to enhance signal interpretation, optimize device fitting, and enable dynamic adaptation to individual users’ neural and behavioral responses [13,14].

Yet, integrating AI into biologically grounded systems raises important open questions. Below, we highlight three illustrative examples designed to stimulate reflection and guide future interdisciplinary research at the interface of computational science, auditory biology, and clinical neurotechnology [13,14]:

- How can AI adapt stimulation strategies in real time based on the user’s neural responses and hearing profile?
  Real-time adaptation could personalize therapy. However, it requires robust biomarkers of auditory performance and interpretable algorithms [13,14].
- Which biological signals (e.g., neural activity, evoked potentials) are most reliable and practical for guiding AI-based decisions in auditory devices?
  Identifying the signals that best capture auditory state is crucial for developing effective feedback systems [13,14].
- To what extent can AI improve long-term outcomes by accounting for neural plasticity and changes in auditory function over time?
  This question links machine learning approaches with the biological reality of a constantly adapting nervous system [13,14].

### 1.3. Aim

In light of rapid advancements in artificial intelligence (AI) and neuroprosthetic technologies, it is important to map the current state of the field through a dedicated review study. Table 1 below summarizes the main types of auditory neuroprosthetic devices, their targets, mechanisms, clinical indications, and AI-related opportunities. The last column highlights potential avenues for investigation in this review, along with other AI-related aspects that may emerge during the review process.

Therefore, this narrative review of reviews aims to provide a comprehensive overview of recent progress in this interdisciplinary field, with three key objectives:

1. Evolution of Scientific Output: To examine publication trends over recent years concerning AI applications in auditory neuroprosthetics, identifying increases in research activity, emerging subfields, and shifts in focus areas.
2. Key Themes and Categorization: To identify and categorize main topics such as AI-driven signal processing, neural interface optimization, and adaptive stimulation strategies across different device types.
3. Opportunities and Challenges: To discuss the practical benefits of AI integration in improving device performance and user outcomes, alongside challenges such as biological variability, data limitations, and ethical considerations.

This narrative approach is particularly suitable given the fast-evolving and complex nature of the field, allowing a flexible yet thorough synthesis that acknowledges both technological innovation and biological intricacies. By focusing on the latest reviews and meta-analyses, this work intends to synthesize existing knowledge and guide future research directions toward smarter, biologically informed neuroprosthetic hearing solutions.

## 2. Methods

This narrative review explores recent developments in the integration of artificial intelligence (AI) with auditory neuroprosthetic devices, focusing on both technological innovations and the underlying neurobiological processes. To ensure comprehensive coverage, the literature search was performed across PubMed, Scopus, and Web of Science databases, prioritizing studies published in the last 5 years. Given the interdisciplinary nature of this review, which focuses on the integration of artificial intelligence (AI) in auditory neuroprosthetic devices, three bibliographic databases were selected: PubMed, Scopus, and Web of Science (WoS). PubMed provides comprehensive coverage of biomedical and clinical literature, ensuring inclusion of studies on cochlear implants, auditory brainstem implants, and related neuroprosthetic interventions. Scopus offers a broader scope, encompassing engineering, computational modeling, and AI applications relevant to signal processing and adaptive device algorithms. Web of Science (WoS) complements these sources by indexing high-impact journals and conference proceedings, allowing tracking of influential studies and emerging trends in neurotechnology and AI.

This combination of databases ensures a robust, interdisciplinary capture of literature spanning clinical, technological, and computational domains. It facilitates identification of both established knowledge and novel research directions in AI-driven auditory neuroprosthetic solutions, while supporting a systematic overview of key themes, methodological approaches, and potential avenues for further investigation

The review focuses on existing review articles rather than individual original studies. Reviews provide high-level summaries of large bodies of research, highlighting key trends, conceptual frameworks, and methodological approaches. This strategy allows the synthesis of complex information while maintaining biological and clinical relevance. It also avoids overemphasizing isolated studies or highly specialized computational work that lacks direct application to auditory neuroprosthetics.

The review focused on studies with a biologically informed perspective, emphasizing auditory pathways, neuroprosthetic mechanisms, and AI-driven interventions. Particular attention was paid to research addressing biological processes, such as neural plasticity, the dynamics of auditory pathways, and the interactions between neuroprosthetic devices and neural tissue.

To ensure relevance and scientific rigor, priority was given to recent publications that cite or build upon foundational work, allowing the review to capture the latest advances while remaining anchored in established knowledge. Conversely, studies that have been superseded, contradicted by more recent evidence, or those focusing solely on algorithmic performance, numerical optimization, or purely computational metrics without clear links to biological or clinical outcomes were generally excluded.

This approach allows the review to synthesize high-priority, current findings, highlight emerging trends, and identify potential avenues for AI integration in auditory neuroprosthetics, while maintaining an integrative perspective that bridges technology and life sciences.

Although narrative reviews do not require rigid inclusion criteria or quantitative scoring, this review followed the a narrative checklist [15] for narrative reviews to ensure methodological transparency, rigor, and comprehensive reporting. This approach balances thoroughness with flexibility, allowing the capture of emerging trends, interdisciplinary insights, and novel conceptual developments in AI-enabled neuroprosthetics.

Unlike systematic reviews, which impose strict inclusion criteria and focus mainly on quantitative synthesis, narrative reviews allow for the exploration of emerging concepts, unresolved challenges, and interdisciplinary opportunities. This approach supports a nuanced understanding of how AI can be translated into biologically informed auditory device innovations.

A composite search key (reported in Box S1 in Supplementary Material) was used to capture the relevant literature comprehensively. Among the terms, a few—such as cochlear implant, auditory brainstem implant, artificial intelligence, machine learning, and neural networks—appeared immediately more closely related to the topic. Additional terms from the composite key were included because preliminary simulations indicated that they also returned relevant outputs before starting the review. Priority was given to recent publications, which consolidate and extend prior foundational work, ensuring that the review reflects the latest advances while acknowledging earlier contributions to the field.

In summary, this review provides a curated, biologically oriented synthesis of the recent literature on AI in auditory neuroprosthetics. It highlights key progress, emerging thematic clusters, remaining challenges, and directions for future research that integrate computational innovation with auditory biology and clinical practice.

## 3. Results

The results of this study are organized into four main sections, each highlighting a distinct aspect of the literature on AI-enabled auditory neuroprosthetics.

Section 3.1 presents the article selection process, reporting the number of studies initially retrieved, screened, included, and excluded, along with the main reasons for exclusion.

Section 3.2 offers a bibliometric analysis, illustrating research trends in the intersection of artificial intelligence and auditory neuroprosthetics and publication patterns over time, revealing the evolution and focus areas of the field.

Section 3.3 provides an interpretative synthesis of the literature, highlighting key messages, emerging themes, and scientific narratives. This section examines, for example, advances in the personalization of auditory implants, integration of electrophysiological biomarkers, predictive modeling of outcomes, and the alignment of AI applications with neurocognitive and perceptual dimensions.

Finally, Section 3.4 discusses the emerging opportunities and challenges identified through the analysis. It emphasizes gaps in current knowledge, strategic directions for future research, and considerations for addressing fragmented biological data, biomarker standardization, regulatory and ethical issues, engineering-centric development, and inequities in access. This section frames the literature within the broader context of AI-driven innovation in auditory neuroprosthetics.

### 3.1. Study Selection Flow (Narrative Review, PRISMA-Inspired Description)

The study selection process followed a narrative review approach, informed by PRISMA principles to ensure transparency in literature identification and inclusion. Literature searches were conducted across three major bibliographic databases: PubMed, Scopus, and Web of Science (WoS), using keywords related to auditory neuroprosthetics, cochlear implants, auditory brainstem implants, hybrid implants, and AI applications in hearing devices.

The initial search retrieved 37 reviews from PubMed, 62 from Scopus, and 48 from WoS, for a total of 147 records. After removing duplicates across databases, 58 unique review articles remained for screening.

Title and abstract screening was then performed to identify studies that met the predefined selection criteria. This step excluded records that were not relevant to biologically informed perspectives, neuroprosthetic mechanisms, or AI integration in auditory devices. After this screening, 47 articles were deemed potentially eligible for full-text assessment.

Full-text assessment was conducted to confirm relevance, methodological rigor, and biological focus. Studies that were superseded, integrated by more recent evidence, or focused solely on computational or algorithmic performance without clear biological or clinical relevance were excluded. This resulted in a final set of 18 reviews included in the integrative synthesis.

This process ensured that the review captures current, high-priority findings, highlights emerging trends, and identifies potential avenues for AI integration in auditory neuroprosthetics, maintaining an integrative perspective bridging technology and life sciences.

### 3.2. Analysis of Publication Trends in the Field of Interest

We conducted a comprehensive analysis of publication trends in auditory neuroprosthetic research in PubMed, comparing AI-focused versus non-AI studies using the search keys in positions 1 and 2 of Box S2 (Supplementary Material). Table 2 summarizes the number of studies, temporal distribution, and proportions of review articles for both categories.

The comparison, as shown in Table 2, reveals several insights:

- The relative growth rate of AI-focused publications exceeds that of non-AI studies, particularly in the last 5 years (39.6% vs. 29.3%), emphasizing AI’s increasing centrality in driving research and translational applications.
- AI-focused reviews remain slightly fewer (6.7% vs. 8.9%), reflecting the novelty and rapid evolution of this subfield, where original studies often precede the consolidation of knowledge in review format.
- Conversely, non-AI research demonstrates more mature progress with established theories, enabling more comprehensive reviews.

Overall, these trends indicate that AI is rapidly becoming a core component of auditory neuroprosthetic research, with a growing body of work highlighting both technological advances and biologically informed applications. These trends underscore AI’s central role in auditory neuroprosthetic research, justifying this narrative review of reviews focused on recent, biologically informed studies bridging neurobiology and AI.

### 3.3. Common Message and Emerging Themes: Interpretation

Eighteen recent review studies [16,17,18,19,20,21,22,23,24,25,26,27,28,29,30,31,32,33] were included, covering all PubMed-indexed reviews from 2019 onward and fully represented in Scopus (Table 3). These works provide a comprehensive synthesis of auditory neuroprosthetic research, highlighting the integration of biology, clinical practice, and AI-driven technology.

Common message: Across these studies, a consensus emerges that successful auditory neuroprosthetic interventions depend on understanding biological mechanisms—neural integrity, auditory pathway dynamics, and patient-specific variability in cochlear nerve function. AI and machine learning are leveraged as tools to interpret complex neural data, predict outcomes, optimize device performance, and tailor rehabilitation strategies [16,17,22]. Innovations in sound encoding, bioelectronic devices, preoperative imaging, tele-audiology, haptic stimulation, and surgical robotics illustrate how technology integrates with biological and clinical insights to improve auditory restoration [18,19,20,21,24,25,26,27,29,30,33].

Some included studies provide broader perspectives on AI and robotic technologies in clinical otology and neurotology [24,26], offering conceptual insights, translational considerations, and illustrative cases that inform potential applications in auditory neuroprosthetic devices.

Emerging themes: Interpretation of biological and clinical data is central to advancing auditory neuroprosthetics, as highlighted by the following patterns:

- AI and Machine Learning for Outcome Prediction: Predictive models rely on biological and neural data to optimize cochlear implant efficacy [16,17,22].
- AI-Driven Device Innovation: Real-time adaptation to biological signals enables personalized and dynamic device performance [18,19].
- Integration in Clinical Practice: Combining biological and clinical data supports informed interpretation and tailored interventions [20,24,25,27].
- Advanced Surgical and Robotic Approaches: AI and robotics facilitate interpretation of anatomical complexity and surgical risk [26].
- Sensory Augmentation and Bioelectronic Platforms: Haptic stimulation, tele-audiology, and self-powered devices enhance interpretation of biological feedback [21,29,30].
- Patient-Specific Neural Assessment: Electrophysiological methods provide detailed interpretation of neural health to guide implant programming [33].

Overall, these reviews highlight how careful interpretation of biological insights, combined with AI and technological innovations, enables predictive modeling, personalized interventions, and novel device design, while suggesting areas for further synthesis and critical evaluation in this rapidly evolving field.

### 3.4. Opportunities and Challenges: Interpretation

The selected review literature [16,17,18,19,20,21,22,23,24,25,26,27,28,29,30,31,32,33] highlights the convergence of artificial intelligence (AI), biological modeling, and clinical innovation in auditory implants, particularly cochlear implants (CIs). This body of work demonstrates how interpretation of biological and neural data can guide predictive modeling, personalized rehabilitation, and device optimization.

Opportunities: Recent studies reveal multiple avenues for innovation. Machine learning enhances outcome prediction and supports individualized rehabilitation, integrating neural plasticity, auditory nerve integrity, and patient-specific cochlear anatomy [16,17,22]. AI-driven signal processing, bioinspired sound encoding, and high-resolution imaging facilitate precise neural targeting and surgical planning [18,19,27,31,32]. Sensory augmentation, haptic feedback, telehealth, and robotic platforms further leverage biological insights to enhance auditory restoration [21,26,29,30]. These emerging opportunities are summarized in Table 4, highlighting the interplay between biology, AI, and clinical practice.

Challenges Despite these advances, several persistent barriers remain to be addressed. Fragmented biological datasets and the absence of standardized biomarkers constrain robust AI interpretation and predictive accuracy [16,17,22,23,25,28,33]. Regulatory and ethical challenges, compounded by the limited explainability of many models, raise concerns about safety, accountability, and patient trust [20,24,25]. Development that remains engineering-centric risks prioritizing technical performance over perceptual, cognitive, and ecological outcomes, while the underrepresentation of non-cochlear devices perpetuates imbalances in innovation [18,19,21,32]. Finally, inequities in access and dataset diversity may exacerbate disparities in outcomes, as AI systems often adapt more effectively to well-represented populations [26,29]. These challenges are synthesized in Table 5, highlighting key areas for future progress in AI-enabled auditory neuroprosthetics.

## 4. Discussion

The discussion interprets the findings of this narrative review, linking evidence synthesis with forward-looking insights on AI integration into auditory neuroprosthetics. Its purpose is twofold: first, to consolidate the knowledge gained from reviewing both mature and emerging domains; second, to map the field’s opportunities, limitations, and strategic directions in light of cutting-edge research. It is organized into six sections.

Section 4.1 presents synoptic graphics that describe the rationale flow and, with particular emphasis on editorial tools such as tables, show the interconnection and development of results and discussion.

Section 4.2 Contribution and Summary of Key Findings highlights the main achievements of AI applications, including predictive modeling, biologically inspired signal processing, and AI-assisted surgical planning, while also identifying emerging opportunities such as multisensory augmentation systems, cross-modal neuroplasticity strategies, and AI-enabled remote care infrastructures.

Section 4.3 Limitations and Gaps, building on the challenges identified in the overview of reviews, this section outlines concrete recommendations to guide future development directions. By addressing key limitations such as fragmented datasets, inconsistent use of biologically meaningful biomarkers, ethical and regulatory complexities, and the limited exploration of non-cochlear auditory prostheses, these recommendations aim to provide actionable guidance for researchers, clinicians, and developers.

Building on these insights, Section 4.4 Discussion in Light of Cutting-Edge Research presents a differential analysis, showing how recent studies attempt to address the previously identified gaps and align with strategic recommendations for advancing AI-enabled auditory neuroprosthetics.

Section 4.5 Perspective and Future Directions then extends the discussion toward future developments, structured across four dimensions: market growth and demographic drivers, AI integration and technological innovation, regulatory and standardization frameworks, and AI-enabled personalized medicine including data integration and adaptive energy management.

Finally, Section 4.6 Limitations of the Study reports how this narrative review offers a timely synthesis of AI integration in biologically grounded auditory neuroprosthetics, while acknowledging potential biases in study selection, interpretive focus, language, and scope. Nevertheless, its thematic and integrative approach provides a flexible framework to capture emerging trends and support ongoing discussion as the field evolves.

Overall, this structure allows the discussion to move seamlessly from summarizing key contributions, through critical analysis of gaps, to forward-looking perspectives and methodological reflection, providing a comprehensive framework to guide research, clinical practice, and innovation in AI-enhanced auditory neuroprosthetics.

### 4.1. Synoptic Diagram

**Figure 1:**
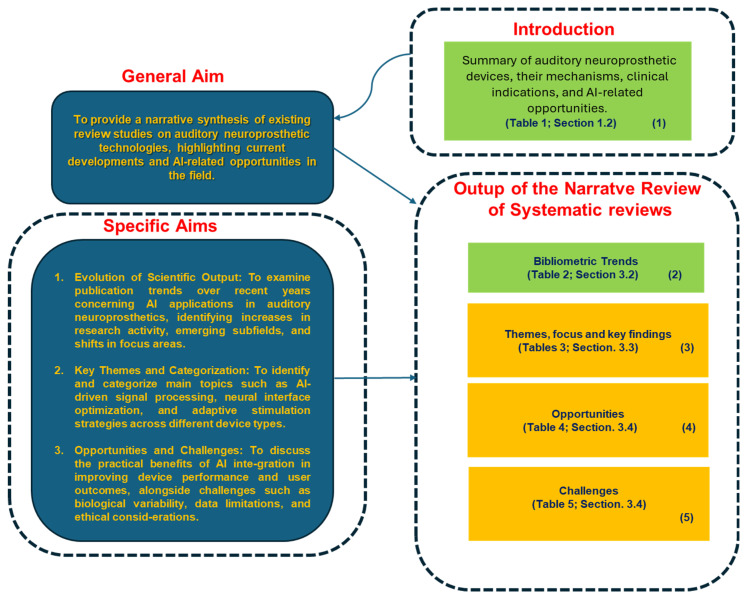
Synoptic diagram of the study structure: from bibliometric trends to AI application in neuroprosthesis.

**Figure 2:**
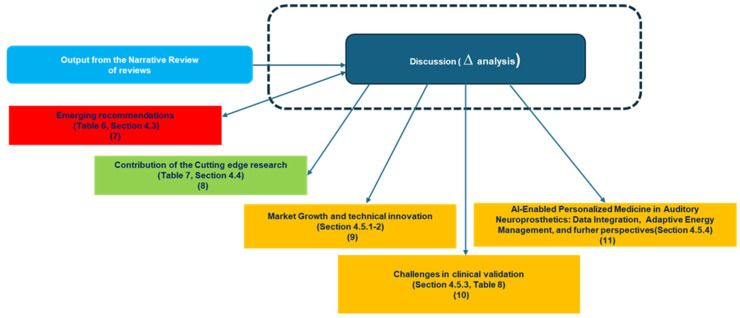
Logical progression from the narrative review findings to the cutting-edge research and perspectives. The triangle indicates the term “differential”.

Figure 1 and Figure 2 present two synoptic diagrams that outline the rationale behind the design of the narrative review. These diagrams provide a structured visual representation of how the study was developed, showing the logical sequence of its different phases and how they interconnect.

#### 4.1.1. First Diagram (Figure 1): Linking Objectives to Analysis

The first diagram (Figure 1) illustrates how the study was structured based on its general objective and three specific objectives. The logical progression follows a top-down approach:

1. Block 1 recalls the introductive summary of auditory neuroprosthetic devices, their mechanisms, clinical indications, and AI-related opportunities reported in Table 1 (Section 1.2).
2. Block 2: This block represents the bibliometric trends reported in Table 1 (Section 3.2). These trends were analyzed to provide an overview of scientific production in the specific field.
3. Block 3: This block corresponds to the identification of thematic areas, as presented in Table 3 (Section 3.3). This categorization allowed for the organization of the reviewed studies according to key themes, focus, and contribution, facilitating a structured analysis.
4. Block 4 and Block 5: Building upon the thematic categorization, these blocks highlights the emerged opportunities as shown in Table 4 (Section 3.4) and open challenges in the field as detailed in Table 5 (Section 3.4)

This diagram provides a step-by-step visualization of the study’s methodological process, from bibliometric analysis to thematic categorization, and the identification of emerging opportunities and challenges.

#### 4.1.2. Second Diagram (Figure 2): Connecting Discussion to Findings

The second diagram (Figure 2) is logically connected to the first and illustrates how the study transitions from the findings of the literature review to the discussion. The sequential organization follows a structured approach:

5. Block 7 identifies the emerging recommendations from the overviewed studies as reported in Table 6 (Section 4.3).

Using these recommendations, the discussion develops incrementally.

6. Block 8 presents the contribution of cutting-edge studies, referring to Table 7, which connects each contribution to the corresponding recommendation.
7. Blocks 9, 10, and 11 address different perspectives: growth and technological implications (Block 9, as discussed in Section 4.5.1 and Section 4.5.2), clinical evaluation (Block 10, as detailed in Table 8, Section 4.5.3), and personalized medicine (Block 11, as covered in Section 4.5.4).

### 4.2. Contribution and Summary of Key Findings

This narrative review of reviews offers a consolidated and biologically anchored synthesis of recent research trends at the intersection of artificial intelligence (AI) and auditory neuroprosthetics. By focusing on published reviews rather than primary studies, the work aims to capture both the maturation of established topics and the conceptual emergence of newer, cross-disciplinary domains. This higher-level perspective enables the identification of methodological convergences, thematic clusters, and gaps that are often obscured when focusing solely on experimental results.

The reviewed literature [16,17,18,19,20,21,22,23,24,25,26,27,28,29,30,31,32,33] underscores how AI is being increasingly integrated into several aspects of auditory neuroprosthetic care, particularly in cochlear implants and related systems. Mature areas include predictive modeling of user outcomes [16,17,22], biologically inspired signal processing [18,19,32], and AI-supported surgical planning [27,31]. These domains reflect a growing synergy between data-driven approaches and core biological principles—such as neural plasticity, cochlear anatomy, and auditory nerve integrity—which has begun to shape both device design and rehabilitation protocols.

Importantly, the review also points to less explored but highly promising areas of research. These include the development of multisensory augmentation systems [21,30], cross-modal neuroplasticity strategies, and AI-enabled remote care infrastructures [26,29]. Such topics remain fragmented across the literature, and their clinical implementation is still in its early stages. Nonetheless, their potential impact—particularly in supporting patient-specific, adaptive, and inclusive solutions—marks them as critical frontiers for future investigation.

By adopting a narrative approach, this review supports a nuanced and flexible mapping of the field, particularly valuable in a domain that evolves rapidly and draws from heterogeneous disciplines. The synthesis presented here does not merely catalog technologies; rather, it clarifies conceptual trajectories and promotes biologically meaningful integrations between AI systems and auditory neural function.

Ultimately, the value of this narrative review lies in its ability to identify stable foundations as well as opportunities for innovation, offering guidance for researchers working across auditory neuroscience, machine learning, and neuroprosthetic design. In doing so, it reinforces the need for research frameworks that remain attentive to biological relevance while embracing computational advancement—a dual imperative that will likely define the next phase of development in this rapidly evolving field.

### 4.3. Limitations and Gaps

Despite significant progress in integrating AI into auditory neuroprosthetics, several limitations persist that constrain both the generalizability and the clinical utility of current approaches.

First, as repeatedly emphasized [16,17,22,25], the field still suffers from fragmented biological and clinical datasets. Models are often trained on retrospective clinical data lacking high-resolution biological indicators such as patterns of neuronal degeneration, cochlear fibrosis, or neuroplasticity profiles. This data sparsity severely limits the ability to personalize auditory neuroprosthetics.

Second, the absence of standardized, biologically meaningful biomarkers—such as eABRs, ECAPs, or ASSRs—hinders the development of interpretable and adaptive AI systems [23,28,33]. While these electrophysiological markers have demonstrated value for auditory monitoring, they are inconsistently used across centers and underreported in algorithm design, reducing reproducibility.

Third, ethical, regulatory, and algorithmic transparency challenges remain pronounced, particularly when AI models interpret neural signals without a clear explainability framework [20,24,25]. This is especially concerning in neuroprosthetic contexts, where model-driven decisions have direct consequences for patients’ sensory experience and neurological development.

A fourth gap lies in the persistent engineering-centric focus of current systems. Many studies report enhancements in signal-to-noise ratios or classification accuracy [18,32], yet few evaluate how these improvements translate to subjective measures such as speech perception under ecological conditions or user satisfaction—factors deeply tied to biological and cognitive responses.

Notably, there is also a visible scarcity of AI-driven studies beyond cochlear implants. Despite the growing literature, little attention has been given to other auditory or vestibular neuroprosthetic devices (e.g., auditory brainstem implants, electro-haptic aids, or hybrid acoustic-electric interfaces), creating an imbalance in innovation spread.

Finally, broader challenges such as health equity, technological access, and underrepresentation of biological diversity in datasets [26,29] persist and risk reinforcing disparities if not actively addressed.

To support the adoption and future development of AI-enhanced auditory neuroprosthetic devices, including but not limited to cochlear implants, the key recommendations derived from the analysis of identified challenges and gaps are summarized in Table 6.

**Table 6:** Recommendations to Address Challenges in AI-Driven Auditory Neuroprosthetics.

#	Challenge	Strategic Recommendation	Ref
1	Fragmented biological data	Promote multi-institutional biorepositories including histological, imaging, and omics data	[16,17,22,25]
2	Lack of standardized biomarkers	Develop consensus guidelines for collection and reporting of ECAPs, eABRs, and ASSRs	[23,28,33]
3	Regulatory and ethical barriers	Co-develop interpretable AI frameworks with biomedical and regulatory stakeholders	[20,24,25]
4	Engineering-centric development	Integrate neurocognitive and perceptual metrics into training and evaluation pipelines	[18,32]
5	Underrepresentation of other devices	Expand AI applications to vestibular implants, auditory brainstem interfaces, etc.	[19,21]
6	Inequity and limited access	Embed ethical frameworks and data diversity requirements in AI development practices	[26,29]

### 4.4. Discussion in Light of Recent Cutting-Edge Research

In light of the limitations and gaps identified through the narrative review of reviews, it becomes not only timely but almost mandatory to explore how recent cutting-edge studies may contribute to addressing these unresolved challenges. This step is a natural progression of the present work, which has highlighted the need to move beyond consolidated evidence and embrace more recent and potentially transformative advances. Following the first key outlined in Box S1 and in line with the adopted methodology, the following studies were selected for further analysis [34,35,36,37,38,39,40,41,42,43,44,45,46,47,48,49,50,51,52,53,54,55,56,57,58,59,60,61,62,63,64,65,66,67,68,69].

These studies provide concrete examples of how recent research aligns with the strategic recommendations for overcoming barriers in AI-enabled auditory neuroprosthetics (Table 7).

The fragmentation and scarcity of biologically rich datasets—a major bottleneck for personalized auditory neuroprosthetics—find some partial alleviation in genetic and anatomical research. For instance, González-Aguado et al. [35] explore Diaphanous Related Formin 1 gene (DIAPH1) mutations linked to sensorineural hearing loss, enriching the biological knowledge base that can inform more tailored AI models. Similarly, investigations into preoperative factors influencing cochlear implant (CI) outcomes [39], and anatomical considerations for individualized implantation [40], further augment biological and clinical data relevant for personalization. However, these efforts remain somewhat siloed and do not yet translate directly into comprehensive multi-modal datasets that can be leveraged for AI training.

The lack of standardized and biologically meaningful biomarkers—such as ECAPs (Electrically Evoked Compound Action Potentials) or eABRs (Electrically Evoked Auditory Brainstem Responses)—that are consistently incorporated into AI algorithms continues to impede interpretability and reproducibility. In this context, Garcia and Carlyon’s work [41] introduces a panoramic ECAP method to assess electrode array differences, advancing biomarker utility and standardization. This represents a meaningful step toward embedding richer electrophysiological signals in AI models, potentially improving their biological grounding and clinical relevance.

Ethical and regulatory concerns around AI transparency and explainability remain pressing, especially given the direct impact of AI decisions on patients’ sensory and neurological outcomes. Here, several studies offer encouraging directions. Icoz and Parlak Kocabay [34] evaluate ChatGPT’s role as an informational tool for cochlear implants, highlighting both potentials and pitfalls of large language models (LLMs) in clinical contexts. More directly related to clinical prediction, Demyanchuk et al. [37] develop a machine learning model to forecast postoperative speech recognition, comparing it rigorously against expert clinical judgment—this juxtaposition is crucial to building trust and explainability in AI applications. Moreover, Jehn et al. [42] utilize convolutional neural networks to improve decoding of selective auditory attention, demonstrating how advanced AI can align closely with cognitive and neural processes, thus enhancing interpretability and functional relevance.

While many AI systems focus heavily on engineering metrics like signal-to-noise ratio or classification accuracy, fewer address real-world subjective outcomes such as speech perception in everyday environments. The CCi-MOBILE (Cochlear Implant Mobile) research platform [36] facilitates real-time sound coding studies, supporting more ecologically valid assessments. Combined with predictive models of speech outcomes [37] and analyses of learning curves post-implantation [39], these studies underscore a growing emphasis on integrating neurocognitive and perceptual metrics alongside traditional engineering benchmarks.

Notably absent from this recent literature is significant progress on expanding AI applications beyond cochlear implants to other auditory or vestibular neuroprosthetic devices, indicating a persistent imbalance in innovation distribution.

Broader social concerns such as health equity and access are only tangentially addressed. Hughes et al. [38] emphasize evidence-based strategies for early detection and management of age-related hearing loss, which indirectly relate to equitable care and access to auditory technologies. However, explicit incorporation of ethical frameworks and data diversity considerations in AI development remains underexplored.

Continuing the analysis of recent literature, several cutting-edge studies appear to move in the direction of overcoming some of the limitations highlighted in earlier research on AI-enabled auditory neuroprosthetics [43,44,45,46,47,48,49,50,51,52,53,54,55], offering promising—though not definitive—contributions. Wohlbauer et al. [43] explore combined channel deactivation and dynamic current focusing strategies to improve speech outcomes in cochlear implant users, moving beyond purely signal-to-noise enhancements towards optimizing perceptual benefits in real-world listening environments. This aligns with the recommendation to integrate perceptual metrics into system evaluations.

Liu et al. [44] propose a novel estimation method for dynamic range parameters based on neural response telemetry thresholds, enhancing individualized parameter setting by leveraging electrophysiological markers, which helps address the lack of standardized, biologically meaningful biomarkers crucial for adaptive AI algorithms.

Müller et al. [45] demonstrate that ambient noise reduction algorithms can reduce listening effort and improve speech recognition in noise among MED-EL cochlear implant users, reinforcing the need to incorporate ecological validity and user-centered outcomes in device optimization.

Shew et al. [46] apply machine learning beyond single biomarkers, incorporating multiple physiological and behavioral features to better predict cochlear implant outcomes. This multidimensional modeling approach directly addresses fragmented data and biomarker standardization challenges, promoting more interpretable and robust AI systems.

Zhao et al. [47] and Sinha et al. [48] highlight the importance of neural communication patterns and early cognitive development prediction in cochlear implant recipients, suggesting that integrating neural connectivity and developmental biomarkers can enhance personalization and long-term functional predictions.

Avallone et al. [49] and Schraivogel et al. [50] focus on automated surgical planning and radiation-free electrode localization through impedance telemetry, advancing engineering aspects toward safer and more precise implantations, which can improve patient-specific outcomes and reduce procedural risks.

Skarżyńska et al. [51] introduce local steroid delivery via an inner ear catheter during implantation, a novel clinical intervention with potential to modulate the biological environment, thus addressing the need for richer biological data integration.

Franke-Trieger et al. [52] develop a voltage matrix algorithm to detect electrode misplacement intraoperatively, enhancing procedural accuracy and implant functionality.

Yuan et al. [53] utilize preoperative brain imaging to predict auditory skill outcomes in pediatric patients, representing a step toward incorporating central nervous system biomarkers and improving prognostic modeling.

Finally, Gnadlinger et al. [54] report on an intelligent tutoring system integrated into game-based auditory rehabilitation, offering a user-centered, adaptive training approach that may improve long-term functional gains and patient engagement.

Together, these studies contribute novel methodologies and clinical tools that advance the field beyond existing limitations: improving biological data richness and standardization, integrating multi-dimensional AI models, enhancing interpretability and explainability, expanding beyond cochlear implants to encompass surgical and rehabilitative domains, and emphasizing patient-centered outcomes and ethical considerations.

Several further recent studies [55,56,57,58,59,60,61,62,63,64,65,66,67,68,69] provide valuable contributions that address persisting gaps or open promising pathways in the field of cochlear implants (CIs), particularly at the intersection of engineering innovation, personalized medicine, and digital health.

Some works aim to enhance intraoperative precision. Babajanian et al. [55] propose a novel algorithm for analyzing multi-frequency electrocochleography data to better monitor electrode placement during surgery, directly responding to the gap in real-time feedback tools. Similarly, Siebrecht et al. [64] and Radomska et al. [65] develop automated segmentation tools and CT-based evaluations that improve anatomical guidance and preoperative planning, bridging technical and anatomical knowledge for improved outcomes.

Regarding safety and comorbidity management, Zarchi et al. [56] raise a critical issue about transcranial stimulation in CI users, calling for clearer safety guidelines in neuromodulation practices. Their case-based approach underlines the need for more nuanced risk assessments in CI populations, especially as neurostimulation techniques expand.

Advances in outcome prediction and personalization are well represented. Wohlbauer et al. [57] explore channel deactivation strategies for speech-in-noise performance, offering a flexible solution that could complement individualized programming. Chen et al. [58] use functional near-infrared spectroscopy to predict pediatric outcomes—expanding the tools available for tailoring expectations and interventions in prelingual deafness. In parallel, Patro et al. [62] validate a machine learning model leveraging multifactorial preoperative data, demonstrating its utility in personalized prognosis, while Sinha and Azadpour [60] assess acoustic simulations through deep learning for real-time speech evaluation. These studies collectively target the gap in reliable, patient-specific outcome modeling.

Technological innovation in CI hardware and software also continues to evolve. Sahoo et al. [61] compare conventional versus AI-enhanced speech processors, showing improvements in audiological outcomes. Deng et al. [63] provide real-world data on the Cochlear Nucleus 7 system, offering a rare post-market evaluation of digital upgrades. Henry et al. [59] explore how speech intelligibility varies based on noise-reduction training masks, enriching the understanding of algorithmic tuning under ecological conditions.

Meanwhile, Yusuf et al. [67] and Aktar Uğurlu & Uğurlu [66] contribute from a neurodevelopmental and bibliometric standpoint, respectively. The former identifies altered neural coupling in congenital deafness, reinforcing the need for early intervention and adaptive stimulation strategies. The latter traces publication trends in hearing loss, revealing shifts in research emphasis and underexplored subdomains—a meta-perspective that informs funding and policy directions.

On the translational side, Cramer et al. [68] describe a reproducible method for aligning CI electrode arrays during insertion trials, which can support standardization in device testing. Finally, Wisotzky et al. [69] highlight the role of XR-based telepresence in surgical training and assistance, pointing to future integration of immersive technologies in CI surgery and education—a field that remains nascent but full of potential.

Together, these studies enrich the multidimensional landscape of cochlear implantation by addressing technical, anatomical, algorithmic, and neurodevelopmental gaps. They emphasize the growing convergence of AI, precision diagnostics, surgical robotics, and immersive environments in redefining patient care and clinical decision-making for auditory prosthetics.

**Table 7:** Recent Studies Addressing Challenges and Strategic Recommendations for AI-enabled Auditory Neuroprosthetics. (# indicate the number of recommendation).

Study	Key Contribution	Linked Strategic Recommendation
[34]	Evaluates ChatGPT as an information source for cochlear implants, assessing both accuracy and reproducibility, highlighting potentials and limitations of large language models (LLMs) in clinical decision-making.	# 3 Regulatory and ethical barriers—emphasizes AI transparency, explainability, and building trust in clinical applications.
[35]	Investigates DIAPH1 gene mutations in patients with sensorineural hearing loss, enriching biological knowledge and enabling more personalized predictive models.	# 1 Fragmented biological data—contributes to multi-institutional datasets for individualized AI modeling.
[36]	Describes the CCi-MOBILE platform enabling real-time sound coding and ecologically valid auditory testing in cochlear implant users.	# 4 Engineering-centric development—integrates neurocognitive and perceptual metrics into AI evaluation pipelines.
[37]	Develops a machine learning model to predict postoperative speech recognition outcomes, validated against expert clinical judgment to enhance model interpretability.	# 3 Regulatory and ethical barriers—supports explainable AI and informed clinical decision-making.
[38]	Provides evidence-based strategies for early detection and management of age-related hearing loss, addressing aspects of access and equity.	# 6 Inequity and limited access—informs equitable access strategies and data diversity considerations.
[39]	Analyzes preoperative factors affecting learning curves in postlingual cochlear implant recipients, informing individualized rehabilitation approaches.	# 1 Fragmented biological data—adds clinically relevant preoperative variables to support AI modeling.
[40]	Discusses anatomical considerations for achieving optimized individualized cochlear implant outcomes.	# 1 Fragmented biological data—enriches anatomical datasets for personalized AI predictions.
[41]	Introduces panoramic ECAP method to assess electrode array differences, advancing biomarker standardization for AI integration.	# 2 Lack of standardized biomarkers—enables consistent electrophysiological signal incorporation into AI algorithms.
[42]	Applies convolutional neural networks to improve decoding of selective auditory attention in cochlear implant users, linking neural activity to perception.	# 4 Engineering-centric development—incorporates neurocognitive and perceptual features into AI evaluation.
[43]	Combines channel deactivation and dynamic current focusing to improve speech outcomes in real-world listening conditions.	# 4 Engineering-centric development—emphasizes perceptual and ecological optimization beyond traditional engineering metrics.
[44]	Proposes dynamic range estimation based on neural response telemetry thresholds to optimize individualized cochlear implant parameters.	# 2 Lack of standardized biomarkers—leverages electrophysiological signals to enhance AI model grounding.
[45]	Demonstrates that ambient noise reduction reduces listening effort and improves speech recognition in noise for MED-EL cochlear implant users.	# 4 Engineering-centric development—prioritizes ecological validity and user-centered outcomes in AI training.
[46]	Uses multiple physiological and behavioral features in ML models to predict cochlear implant outcomes, moving beyond single biomarkers.	# 1 Fragmented biological data and # 2 Lack of standardized biomarkers—supports robust, multidimensional AI modeling.
[47]	Highlights asymmetric inter-hemisphere neural communication contributing to early speech acquisition in toddlers with cochlear implants.	# 1 Fragmented biological data—integrates neural connectivity and developmental biomarkers for AI personalization.
[48]	Predicts long-term cognitive and verbal development in prelingual deaf children using six-month performance assessments.	# 1 Fragmented biological data—incorporates early performance measures to improve individualized predictive models.
[49]	Investigates automated cochlear length and electrode insertion angle predictions for surgical planning, optimizing patient-specific implantation.	# 4 Engineering-centric development—enhances safety, precision, and outcome optimization in AI-assisted planning.
[50]	Develops radiation-free localization of cochlear implant electrodes using impedance telemetry, improving intraoperative accuracy.	# 4 Engineering-centric development—improves procedural safety and integration of engineering and clinical metrics.
[51]	Explores local steroid delivery to the inner ear during cochlear implantation to modulate the biological environment.	# 1 Fragmented biological data—provides additional biologically relevant data for AI-based predictive modeling.
[52]	Introduces a voltage matrix algorithm to detect cochlear implant electrode misplacement intraoperatively, enhancing procedural safety.	# 4 Engineering-centric development—integrates precise intraoperative monitoring for improved outcomes.
[53]	Uses preoperative brain imaging to predict auditory skill outcomes after pediatric cochlear implantation.	# 1 Fragmented biological data—enriches datasets with neuroimaging biomarkers for personalized AI predictions.
[54]	Incorporates an intelligent tutoring system into game-based auditory rehabilitation, validating AI-assisted training for adult CI recipients.	# 4 Engineering-centric development—integrates cognitive and perceptual metrics into rehabilitation-focused AI systems.
[55]	Develops a multi-frequency electrocochleography algorithm to monitor electrode placement during cochlear implant surgery.	# 2 Lack of standardized biomarkers—standardizes intraoperative electrophysiological measures for AI integration.
[56]	Case study evaluating safety implications of cochlear implants with transcranial stimulation.	# 3 Regulatory and ethical barriers—informs safety and ethical considerations in AI-guided procedural decisions.
[57]	Evaluates speech-in-noise performance using combined channel deactivation and dynamic focusing in adults with cochlear implants.	# 4 Engineering-centric development—emphasizes perceptual and real-world outcome optimization.
[58]	Predicts individualized postoperative cochlear implantation outcomes in children using functional near-infrared spectroscopy (fNIRS).	# 1 Fragmented biological data—incorporates neuroimaging and physiological biomarkers for AI personalization.
[59]	Investigates the impact of mask type on speech intelligibility and quality in cochlear implant noise reduction systems.	# 4 Engineering-centric development—integrates user-centered metrics to improve AI-assisted sound processing.
[60]	Employs deep learning to evaluate speech information in acoustic simulations of cochlear implants.	# 4 Engineering-centric development—leverages AI to optimize signal processing and perceptual fidelity.
[61]	Compares audiological outcomes between conventional and AI-upgraded cochlear implant processors.	# 4 Engineering-centric development—demonstrates AI-enhanced device performance for clinical relevance.
[62]	Uses machine learning and multifaceted preoperative measures to predict adult cochlear implant outcomes in a pilot study.	# 1 Fragmented biological data and # 2 Lack of standardized biomarkers—integrates multidimensional preoperative data into AI models.
[63]	Real-world evaluation of improved sound processor technology (Cochlear Nucleus 7) in Chinese CI users.	# 4 Engineering-centric development—supports AI-enhanced device optimization with clinical outcome validation.
[64]	Automates segmentation of clinical CT scans of the cochlea, analyzing vertical cochlear profile for implantation planning.	# 1 Fragmented biological data—provides anatomical datasets to enhance AI-guided surgical predictions.
[65]	Evaluates round window access using CT imaging for cochlear implantation.	# 1 Fragmented biological data—adds precise anatomical measurements for AI-assisted surgical planning.
[66]	Bibliometric exploration of hearing loss publications over four decades to identify research trends and gaps.	# 6 Inequity and limited access—informs strategic planning and equitable distribution of AI research resources.
[67]	Shows that congenital deafness reduces alpha-gamma cross-frequency coupling in auditory cortex.	# 1 Fragmented biological data—contributes neurophysiological biomarkers for AI-based predictive modeling.
[68]	Presents a method for accurate and reproducible specimen alignment for CI electrode insertion tests.	# 4 Engineering-centric development—ensures standardized experimental setups for AI training and validation.
[69]	Explores telepresence and extended reality for surgical assistance and training.	# 4 Engineering-centric development and # 6 Inequity and limited access—improves training access and enhances AI-assisted surgical guidance.

### 4.5. Perspective and Future Directions

The future of auditory neuroprosthetics lies at the intersection of growing demand, technological innovation, and stringent regulatory frameworks. This section explores six key dimensions that will shape the evolution of the field: market growth and demographic drivers, the integration of artificial intelligence, regulatory and standardization frameworks, data-sharing platforms with privacy-preserving approaches, and enhanced signal processing and innovative energy-supply systems (alongside obviously continuously advanced technological developments such as miniaturization, improved materials). Each pillar underscores the challenges, opportunities, and strategic directions necessary for the development of neuroprosthetics that are more effective, safer, and sustainable, while pushing the boundaries of human–machine integration.

#### 4.5.1. Market Growth and Demographic Drivers

In outlining the future of auditory neuroprosthetics, three interrelated dimensions emerge as critical: the strong and sustained market growth driven by demographic and technological factors; the crucial role of AI integration supported by evolving regulatory and standardization frameworks; and the inherent unpredictability of AI advancements that demands ongoing vigilance and adaptive governance.

The auditory neuroprosthetics market is experiencing significant growth and is projected to continue expanding over the coming years. According to Mordor Intelligence, the global neuroprosthetics market is expected to reach USD 13.43 billion in 2025 and grow at a compound annual growth rate (CAGR) of 11.83%, reaching USD 23.49 billion by 2030 [70]. Similarly, the Business Research Company forecasts the market size to be USD 11.72 billion in 2025, with a revenue forecast of USD 20.61 billion by 2034, reflecting a CAGR of 15.2% [71]. This growth is driven by several factors, including the increasing prevalence of hearing impairments, advancements in implantable technologies, and a growing aging population. As the demand for effective auditory solutions rises, there is a corresponding need for innovative technologies to enhance the performance and adaptability of auditory neuroprosthetics.

#### 4.5.2. AI Integration and Technological Innovation

Artificial intelligence (AI) is poised to play a pivotal role in this evolution. AI algorithms enable personalized sound processing, real-time adaptation to environmental changes, and improved user outcomes by learning from individual auditory experiences. The integration of AI into auditory neuroprosthetics is expected to lead to more sophisticated and effective devices, further driving market growth and adoption. For example, the market intelligence report by Grand View Research projects a compound annual growth rate (CAGR) exceeding 10% for the neuroprosthetics market through 2030 [72]. This is largely propelled by advances in AI and machine learning that improve device functionality and patient personalization.

It is therefore essential to move toward the routine integration of AI technologies within auditory neuroprosthetic systems. This requires not only technical innovation but also robust regulatory and standardization frameworks to ensure safety, interoperability, and ethical alignment.

#### 4.5.3. Regulatory and Standardization Landscape: Challenges in Clinical Validation

The integration of artificial intelligence (AI) into neuroprosthetic devices increasingly intersects with evolving regulatory frameworks and international standardization efforts, shaping the development of highly reliable, safe, and ethically robust technologies. Global regulatory agencies are actively delineating approaches to ensure AI-enabled medical devices meet stringent requirements for clinical safety, performance validation, transparency, and accountability. Within the European Union, the Medical Device Regulation (MDR) 2017/745 [73] establishes rigorous standards for clinical evaluation, post-market surveillance, and risk management throughout the device lifecycle, while the recently adopted AI Act [74] introduces a risk-stratified framework—commonly termed the “double traffic light” model—classifying AI systems according to potential patient, provider, and societal risk. These frameworks are particularly critical in high-stakes medical domains, including auditory neuroprosthetics, where algorithmic errors or systemic bias can directly impact neural interfacing and auditory perception.

International standardization initiatives provide technical and procedural guidance for trustworthy AI implementation in clinical contexts. ISO/IEC standards—including ISO/IEC 22989 on AI terminology [75], ISO/IEC 23894 on AI system risk management [76], and ISO 81001-1 on health software safety [77]—define best practices encompassing algorithm validation, data integrity, model robustness, reproducibility, interpretability, and continuous post-deployment monitoring. Complementary standards, such as IEC 60601-1-11:2015+A1:2020 [78] on electrical safety and essential performance for medical devices in home healthcare environments, and ISO/IEC 27001:2022 [79] on information security management, further mitigate operational and cybersecurity risks inherent to AI-driven medical devices. These standards collectively facilitate consistent, transparent AI behavior, minimizing adverse outcomes, enhancing clinical interpretability, and enabling interoperability across healthcare infrastructures.

AI integration with personalized medicine in auditory neuroprosthetics poses specific challenges for clinical validation. Algorithms must adapt to individual auditory profiles, neural plasticity, and real-world environmental variability, demanding rigorous testing across diverse patient populations. Standardized frameworks enable structured clinical evaluation, multicenter trials, cross-institutional data aggregation, and generalization of AI models, thereby supporting reliable personalization strategies while maintaining patient safety and reproducibility. Moreover, ethical principles—including patient consent, fairness, privacy, and accountability—are embedded in regulatory measures, fostering public trust and responsible adoption in clinical practice.

The harmonization of regulatory and standardization measures underpins patient safety and catalyzes innovation. Clear requirements for algorithmic performance, data governance, and clinical validation encourage the deployment of advanced AI algorithms, adaptive signal processing systems, and real-time personalization strategies essential for auditory rehabilitation. Given the rapid evolution of AI methodologies, these frameworks must adopt iterative, adaptive governance approaches, incorporating novel algorithms, emerging data modalities, and real-world evidence to ensure sustained efficacy, safety, and ethical compliance. In this context, the interplay of regulation, technical standardization, and ethical oversight serves both as a safeguard and as a strategic enabler, allowing AI-enabled auditory neuroprosthetics to achieve maximal therapeutic impact while advancing patient-centered outcomes.

Table 8 presents a summary of the cited regulatory frameworks. Other relevant standards not specific to AI, such as ISO 13485 (quality management), ISO 14971 (risk management), IEC 62304 (medical device software lifecycle), and diverse others, also apply but are not included here for brevity.

**Table 8:** Key Regulatory and Standardization Frameworks for AI Auditory Neuroprosthetic Devices.

Framework/Standard	Main Scope	Key Objectives	Impact on Auditory Neuroprosthetic Devices	Devices Affected	Practical AI Implementation Examples
MDR (EU Medical Device Regulation) 2017/745	Medical device regulation	Clinical safety, performance, risk assessment, post-market surveillance	Ensures safety and performance of AI-enabled auditory neuroprostheses	CI, ABI, AMI, HAT, BAHS, experimental devices	Pre-market approval; continuous monitoring of device performance and patient outcomes
AI Act (EU)	AI system regulation	Risk-based classification (“double traffic light” with the MDR), transparency, accountability	Regulates high-risk AI algorithms, ensuring robustness, reliability, and ethical deployment in AI-enabled auditory devices	CI, ABI, AMI, HAT, BAHS, experimental devices	Adaptive AI sound processing and neural stimulation algorithms; ethical deployment in high-risk devices
ISO/IEC 22989	AI terminology	Standardization of AI terms and concepts	Provides common language for describing AI functions and models across devices	CI, ABI, AMI, HAT, BAHS	Shared terminology for neural signal processing, ML models for auditory scene analysis
ISO/IEC 23894	AI risk management	Guidelines on identifying, assessing, and mitigating AI-related risks	Supports evaluation of AI reliability, failure modes, and clinical safety	CI, ABI, AMI, HAT, BAHS, experimental devices	Risk assessment for predictive models controlling stimulation patterns
ISO 81001-1	Health software	Safety, quality, lifecycle management of healthcare software	Ensures safe software development and deployment for AI-enabled devices	CI, ABI, AMI, HAT, BAHS	Safe AI firmware updates, software maintenance, patient safety
IEC 60601-1-11:2015+A1:2020	Electrical safety	Protection from electrical hazards	Guarantees electrical safety for AI-integrated auditory devices in home healthcare environments	CI, ABI, AMI, HAT, BAHS	Integration of AI-controlled signal processors and electrode arrays ensuring electrical safety
ISO/IEC 27001:2022	Information security	Secure data management	Protects patient data used for training, calibration, and operation of AI systems	CI, ABI, AMI, HAT, BAHS, experimental devices	Secure cloud-based federated learning, privacy-compliant data sharing

Note: CI = Cochlear Implants; ABI = Auditory Brainstem Implants; AMI = Auditory Midbrain Implants; HAT/ALD = Hearing Aid/Assistive Listening Devices; BAHS = Bone-Anchored Hearing Systems.

#### 4.5.4. AI-Enabled Personalized Medicine in Auditory Neuroprosthetics: Data Integration, Adaptive Energy Management, and Future Perspectives

The development of AI-driven auditory neuroprosthetics critically depends on access to large, high-quality, and diverse datasets, which enable models to generalize across patient populations while supporting personalized medicine interventions. Secure and standardized data-sharing platforms allow AI algorithms to learn from multi-modal datasets—including auditory thresholds, speech perception tests, cognitive assessments, imaging studies, and device usage patterns—while ensuring patient privacy, forming the backbone of personalized auditory medicine.

For example, the Auditory Implant Initiative (Aii) promotes collaboration among cochlear implant centers. Its HERMES (HIPAA-Secure Encrypted Research Management Evaluation Solution) database securely stores de-identified patient information such as hearing history, demographics, and surgical details, enabling aggregated analyses that enhance AI model reliability and support individualized device programming aligned with personalized medicine principles [80]. Similarly, multimodal research platforms at the Hospital Universitario Virgen Macarena in Seville systematically collect interdisciplinary patient data, providing rich inputs for AI models that adapt stimulation parameters to personalized auditory profiles [81].

Federated learning offers a promising approach for privacy-preserving AI development, allowing models to be trained across decentralized datasets without centralizing sensitive patient information [82]. Complementary techniques, including homomorphic encryption (enabling computations on encrypted data) and differential privacy (introducing statistical noise to prevent re-identification), further safeguard confidentiality while maintaining model generalizability, ensuring that personalized medicine approaches can scale safely across institutions [83].

Integrating these AI capabilities with personalized medicine enables dynamic adaptation of neuroprosthetic devices to each patient’s auditory characteristics, neural plasticity, and real-world environments. AI algorithms can continuously optimize sound-processing strategies, electrode stimulation patterns, and signal amplification based on longitudinal patient data, moving devices beyond static, “one-size-fits-all” programming toward truly patient-tailored auditory rehabilitation and fully realizing the goals of personalized medicine.

A complementary avenue for personalization lies in AI-enhanced energy management. Current neuroprostheses, including cochlear implants, rely on external power sources, limiting autonomy and continuous stimulation. Recent advances in biomechanical-to-electrical energy harvesting using inorganic dielectric materials (IDMs) with engineered micro-/nanoarchitectures offer pathways toward self-powered or hybrid-powered devices, enabling personalized energy delivery that adapts to the unique physiological and auditory profiles of each patient [84,85].

By combining personalized AI control, adaptive energy harvesting, and real-time physiological feedback, next-generation auditory neuroprostheses are poised to deliver autonomous, efficient, and patient-centered performance, reducing the burden of device maintenance and enhancing quality of life, fully embracing the principles of personalized medicine.

In a broader perspective, these developments illustrate how AI-enabled, data-driven, and adaptive neuroprosthetic systems can form the foundation of personalized medicine in auditory care, where device programming, stimulation, and energy management are dynamically tailored in real time to the unique characteristics, preferences, and physiological responses of each patient [86,87]. Beyond auditory rehabilitation, this framework provides a blueprint for integrating multimodal patient data—including cognitive, vestibular, and behavioral metrics—into adaptive AI-driven systems, enabling holistic patient-centered interventions [88].

Future directions include the integration of predictive analytics and clinical decision support, where AI models can anticipate individual patient needs, optimize rehabilitation protocols, and guide clinicians in personalized medicine adjustments. Coupled with remote monitoring and telemedicine platforms, these systems allow continuous feedback loops, enhancing real-world applicability and supporting longitudinal personalization even outside clinical settings [86].

Moreover, combining AI-driven neuroprosthetics with genomic, pharmacological, and lifestyle data opens the possibility for precision auditory medicine to interface with broader personalized medicine strategies, aligning device interventions with patient-specific biological and environmental factors. Adaptive learning algorithms can continuously refine device performance based on longitudinal patient trajectories, neural plasticity, and real-world auditory experiences, further enhancing both efficacy and safety [87,88].

Ultimately, these AI-enabled frameworks exemplify how personalized medicine can extend from individualized device control to a comprehensive, integrative approach, bridging cutting-edge technology with truly patient-tailored clinical care. They establish a pathway toward next-generation precision healthcare, where dynamic adaptation, predictive intelligence, and multi-domain integration maximize therapeutic outcomes, patient satisfaction, and quality of life.

### 4.6. Limitations of the Study

While this narrative review does not aim to be exhaustive, its design offers a valuable and timely synthesis of how artificial intelligence is being integrated into biologically grounded auditory neuroprosthetics. Several methodological considerations—sources of potential bias—can inform future developments, yet many of these features are also sources of strength in rapidly evolving fields.

Firstly, the review is thematic-oriented rather than strictly systematic, which introduces selection bias, since studies were chosen based on relevance to key biological and technological themes. Moreover, being narrative, the review could incorporate recent studies incrementally, providing a discussion that evolves with new findings and insights—an advantage in fields undergoing rapid development.

Secondly, the narrative approach involves a degree of interpretive subjectivity, potentially emphasizing certain findings over others. However, this subjectivity also allows for interdisciplinary insights, connecting neural, clinical, and engineering perspectives, and highlighting patterns that a purely quantitative synthesis might miss.

Thirdly, the review focused on English-language publications, which could introduce language bias, but this ensured consistency in interpretation and facilitated the integration of complex biological and technological concepts.

Finally, the emphasis on biologically grounded studies—such as neural coding, plasticity, and auditory pathway dynamics—excluded purely technical or algorithmic research without a clear biological anchor, representing a scope bias. Nevertheless, this targeted approach enhances translational relevance, making the review especially informative for readers interested in clinical and neuroscientific applications.

Overall, while the narrative design introduces some potential biases, it also maximizes the review’s utility by being thematically rich, flexible, and integrative, capable of capturing emerging trends and insights, and supporting incremental discussion as new studies are published.

## 5. Conclusions

This narrative review of reviews brings together current knowledge at the intersection of artificial intelligence and auditory neuroprosthetics, emphasizing biologically grounded innovations and emerging interdisciplinary trends. AI is reshaping auditory devices—particularly cochlear implants—by enabling personalized sound processing, predictive modeling, and improved surgical planning.

Yet, despite these advances, significant challenges remain. Biological data are often fragmented, biomarkers are not standardized, and AI models can be difficult to interpret in clinical contexts. Ethical and regulatory uncertainties add further complexity, limiting the seamless adoption of AI-driven neuroprosthetics.

Looking ahead, research should focus on concrete directions that address these bottlenecks. Harmonizing and standardizing clinical and biological datasets will be essential for building robust AI models, while expanding AI applications to vestibular and other auditory prostheses can broaden the impact of these technologies. At the same time, interpretable and biologically informed AI models must be developed to ensure that predictions can guide personalized therapy and surgical decisions.

Technological advances, such as miniaturization, improved materials, and innovative energy-harvesting systems, will play a key role in making devices more sustainable, comfortable, and widely usable. Equally important is addressing health equity: AI systems must be trained on diverse datasets and designed inclusively to avoid reinforcing disparities. Finally, aligning research with evolving regulatory frameworks will help ensure that innovations are safe, effective, and ready for clinical translation.

In sum, these challenges and opportunities highlight the need for multidisciplinary collaboration, combining engineering, neuroscience, clinical expertise, and ethics. By following these research directions, the field can move toward more effective, safe, and personalized auditory neuroprosthetics, harnessing the full transformative potential of AI while remaining grounded in biological relevance and patient needs.

## Figures and Tables

**Table 1:** Summary of Auditory Neuroprosthetic Devices, Their Mechanisms, Clinical Indications, and AI-Related Investigative Potential Opportunities.

Device Type	Target Structure	Mechanism of Action	Clinical Indication	Key Features/Notes	AI Potential Opportunities/Investigative Focus	References
Cochlear Implant (CI)	Cochlea/Spiral Ganglion Neurons	Converts acoustic signals into electrical impulses delivered via electrode array	Severe-to-profound sensorineural hearing loss with functional auditory nerve	Widely used; preserves frequency resolution; enables auditory information to reach brainstem	Optimization of real-time signal processing, personalized fitting, dynamic adaptation to neural responses	[1,2,3,4,5,9,13,14]
Auditory Brainstem Implant (ABI)	Cochlear nucleus in brainstem	Direct electrical stimulation of cochlear nucleus	Patients lacking functional auditory nerve (e.g., neurofibromatosis type II, tumor resection)	Outcomes generally less favorable than CI; bypasses cochlea and auditory nerve	Electrode mapping, prediction of optimal stimulation patterns, enhancement of speech perception outcomes	[5,6,10,13,14]
Hybrid Cochlear Implant	Cochlea (residual hearing + damaged regions)	Combines natural hearing for low frequencies with electrical stimulation for high frequencies	Partial hearing loss with preserved apical cochlear function	Optimizes residual hearing; integrates biological input with neurostimulation	Adaptive balancing of electrical vs. natural input, optimization of frequency allocation	[5,11,13,14]
Next-Generation Neuroprostheses	High-density electrode arrays/cortical targets	Advanced stimulation strategies, including optogenetic interfaces and AI-driven adaptation	Experimental/research use	Aims to enhance frequency resolution, speech perception in noise, and biocompatibility	Real-time adaptation, prediction of neural plasticity, multi-modal signal interpretation, personalized therapy	[12,13,14]

**Table 2:** Comparative Analysis of Publication Trends in Auditory Neuroprosthetic Research: AI-Focused vs. Overall Literature.

Metric	AI-Focused Auditory Neuroprosthetic Research (PubMed, Search Key 1)	Overall Auditory Neuroprosthetic Research (PubMed, Search Key 2)	Interpretation/Key Observation
Total studies since first record	540 (since 1989)	17,641 (since 1973)	AI-focused research is newer and smaller in absolute volume.
Number of reviews	36 (6.7%)	1573 (8.9%)	Fewer AI-focused reviews reflect rapid evolution and emerging nature of the field.
Studies published last 10 years	364 (67.4%)	9137 (51.8%)	Recent surge in AI-related publications, faster growth than overall field.
Studies published last 5 years	214 (39.6%)	5172 (29.3%)	AI is increasingly central; growth rate exceeds overall field.
Trend observation	Rapid increase in AI publications, especially last 5 years	Slower relative growth; more mature and established research	Highlights AI as a key driver of innovation; overall field shows consolidated knowledge.

**Table 3:** Snapshot of Biological and AI Advances in Auditory Neuroprosthetics Based on the Overviewed Studies.

Reference	Theme	Study Focus/Topic	Biological/Clinical Aspect	Artificial Intelligence/Technological Contribution
[16]	Machine Learning Prediction Models	Systematic review of machine learning approaches to predict outcomes of cochlear implants	Analysis of patient auditory outcomes and variability in hearing restoration after implantation	Evaluation and comparison of various machine learning algorithms aimed at improving outcome prediction and personalizing therapy
[17]	Machine Learning Prediction Models	Systematic review on predicting auditory performance in cochlear implant patients using machine learning	Focus on clinical measures such as speech perception and hearing threshold improvements	Application of supervised machine learning models to improve predictive accuracy for clinical outcomes
[18]	Artificial Intelligence Innovations in Device Design	Overview of artificial intelligence-enabled advances in cochlear implant technologies for hearing restoration	Discussion of auditory prosthesis improvements and patient adaptation	Use of artificial intelligence for adaptive signal processing and optimization of sound quality
[19]	Signal Processing Methods	Review of diverse encoding strategies for sound signals in cochlear implants	Investigation of neural coding and auditory nerve stimulation techniques	Survey of computational encoding algorithms to enhance speech comprehension in implant users
[20]	Artificial Intelligence Applications in Ear, Nose, and Throat Medicine	Current trends and applications of artificial intelligence in the diagnosis and treatment of ear, nose, and throat disorders	Clinical relevance to patient diagnosis and management in otology and related fields	Use of artificial intelligence for diagnostic assistance, treatment planning, and clinical workflow improvements
[21]	Bioelectronics and Self-Powered Devices	Review of piezoelectric materials as self-powered bioelectronic devices	Potential applications in implantable medical devices including auditory prostheses	Discussion of bioelectronic devices using energy harvesting for improved patient device autonomy
[22]	Machine Learning Prediction Models	Systematic review of machine learning prediction models for cochlear implant functional outcomes	Focus on functional hearing outcomes and patient variability	Comparative analysis of machine learning models to improve clinical decision support
[23]	Speech Recognition and Rehabilitation	Review of speech recognition and speech audiometry parameters in assessing rehabilitation progress in cochlear implant patients	Clinical evaluation of auditory rehabilitation effectiveness	Use of speech parameter analysis for monitoring patient progress and tailoring rehabilitation protocols
[24]	Artificial Intelligence in Ear and Neural Surgery	Review of artificial intelligence applications in otology and neurotology surgeries	Impact on surgical planning, outcomes, and patient safety	Use of AI-assisted surgical navigation and intraoperative decision-making tools
[25]	Machine Learning in Otology	Application of machine learning methods to otologic clinical problems	Clinical outcomes and diagnostic improvements	Use of machine learning to improve diagnosis and treatment personalization in ear diseases
[26]	Autonomous Robotic Surgery	Review of autonomous robotic surgery technologies and future prospects	Surgical precision and patient safety in otologic and other surgeries	Development and clinical application of autonomous robotic systems in surgery
[27]	Imaging for Cochlear Implant Rehabilitation	Use of advanced imaging techniques prior to cochlear implantation to improve rehabilitation	Anatomical assessment and surgical planning for implants	Integration of imaging data into preoperative planning and outcome prediction
[28]	Post-Implantation Complications	Study of emphysema occurrence following cochlear implantation, risk factors and treatment	Clinical management of post-surgical complications in implant patients	Discussion of diagnostic and therapeutic options to manage implant-related complications
[29]	Tele-Audiology	Review of current state and future directions of telemedicine applied to audiology	Remote patient monitoring and hearing assessment	Use of digital health tools and telecommunication technologies for hearing care delivery
[30]	Haptic Stimulation in Hearing Impairment	Exploration of haptic stimulation to enhance music perception in hearing-impaired listeners	Sensory integration and auditory perception enhancement	Development of haptic devices and multisensory integration techniques for improved music enjoyment
[31]	Cochlear Implant Research and Development	Critical update on recent research and development in cochlear implant technology	Advances in implant design and auditory neuroscience	Overview of engineering, signal processing and neural interface improvements
[32]	Noise Reduction in Signal Processing	Review of noise reduction techniques in cochlear implant signal processing	Impact on speech understanding in noisy environments	New algorithms and approaches for improving signal-to-noise ratio in implants
[33]	Patient-Specific Neural Health Assessment	Development of the Panoramic Evoked Compound Action Potential method to estimate current spread and neural health	Personalized assessment of auditory nerve status	Use of electrophysiological measurements to tailor cochlear implant settings

**Table 4:** Emerging Opportunities in Auditory Neuroprosthetics.

Opportunity Area	Key Insights	Representative References
Predictive Modeling and Personalized Rehabilitation	Machine learning models enhance prediction of auditory outcomes, support preoperative selection, and tailor rehabilitation; integrates neural plasticity, auditory nerve integrity, and patient-specific cochlear anatomy.	[16,17,22]
AI-Enhanced Signal Processing and Bioinspired Encoding	AI refines signal processing and sound encoding strategies, mimicking cochlear transduction and supporting real-time adaptation to biological data.	[18,19,32]
Imaging, Surgical Planning and Neural Targeting	High-resolution imaging and electrophysiological mapping improve electrode placement and stimulation strategies; targets residual auditory fibers for personalized implant parameters.	[27,31]
Sensory Augmentation and Cross-Modal Plasticity	Multisensory integration, haptic feedback, and visual-auditory compensation leverage cortical plasticity, supporting perception via alternative neural pathways when cochlear input is incomplete.	[21,30]
Digitalization of Care: Telehealth and Robotics	AI-driven remote diagnostics, surgical robotics, and continuous monitoring improve efficiency and accessibility; depends on patient biological response (e.g., ECAP-guided tuning).	[26,29]

**Table 5:** Key Challenges in AI Integration for Auditory Neuroprosthetics.

Challenge Area	Key Insights	Representative References
Fragmented Biological Data	Current datasets often lack critical biological variables (e.g., neural survival, cochlear fibrosis, neurodevelopmental markers), reducing robustness and personalization potential of AI models.	[16,17,22,25]
Lack of Standardized Biomarkers	Absence of consistent and biologically meaningful electrophysiological markers (e.g., ECAPs, eABRs, ASSRs), together with heterogeneous acquisition protocols, limits reproducibility and real-time AI decision-making.	[23,28,33]
Regulatory and Ethical Barriers	Lack of clear frameworks and limited explainability of many AI models pose concerns about safety, accountability, and patient trust, especially when algorithms influence neural-level interventions.	[20,24,25]
Engineering-Centric Development	Development remains largely technology-driven, emphasizing engineering metrics (e.g., SNR, classification accuracy) over perceptual, cognitive, and ecological outcomes essential for human-centered care.	[18,32]
Underrepresentation of Other Devices	Innovations are concentrated on cochlear implants, with vestibular and other auditory neuroprostheses receiving minimal research attention, leaving the field unevenly advanced.	[19,21]
Inequity and Limited Access	Socioeconomic and geographical disparities, coupled with underrepresentation in datasets, risk exacerbating inequities in outcomes, as AI tools may disproportionately benefit well-represented populations.	[26,29]

## Data Availability

Not applicable.

## Supplementary Materials

The following supporting information can be downloaded at: https://www.mdpi.com/article/10.3390/biology14091309/s1, Box S1. The composite key used in the dedicated search; Box S2. The composite key used in the dedicated searches.
