# Supplementary material for: Bridging Neurobiology and Artificial Intelligence: A Narrative Review of Reviews on Advances in Cochlear and Auditory Neuroprostheses for Hearing Restoration
Source: Biology (Basel). 2025 Sep 22;14(9):1309. doi: 10.3390/biology14091309 (PMC12467634; doi:10.3390/biology14091309)
Supplement: Supplementary file 1 [file biology-14-01309-s001.zip › biology-3807633-supplementary.pdf]

**Box S1.** The composite key used in the dedicated search.

*((cochlear implant[Title/Abstract]) OR*
*(cochlear implantation[Title/Abstract]) OR*
*(cochlear implant neural stimulation[Title/Abstract]) OR*
*(cochlear implant signal processing[Title/Abstract]) OR*
*(cochlear implant neural interface[Title/Abstract]) OR*
*(cochlear implant outcomes[Title/Abstract]) OR*
*(cochlear implant auditory pathway plasticity[Title/Abstract]) OR*
*(auditory brainstem implant[Title/Abstract]) OR*
*(ABI implantation[Title/Abstract]) OR*
*(auditory brainstem implant outcomes[Title/Abstract]) OR*
*(auditory brainstem implant neural stimulation[Title/Abstract]) OR*
*(auditory brainstem prosthesis[Title/Abstract]) OR*
*(brainstem auditory prosthesis[Title/Abstract]) OR*
*(advanced auditory neuroprosthesis[Title/Abstract]) OR*
*(auditory neuroprosthetic devices[Title/Abstract]) OR*
*(neural auditory prosthesis[Title/Abstract]) OR*
*(implantable auditory neuroprosthesis[Title/Abstract]) OR*
*(neuroprosthetic auditory stimulation[Title/Abstract]) OR*
*(next generation auditory neuroprostheses[Title/Abstract]) OR*
*(hybrid cochlear implant[Title/Abstract]) OR*
*(electro-acoustic stimulation cochlear implant[Title/Abstract]) OR*
*(combined acoustic and electric hearing[Title/Abstract]) OR*
*(hybrid auditory prosthesis[Title/Abstract]))*
AND
*((artificial intelligence[Title/Abstract]) OR*
*(machine learning[Title/Abstract]) OR*
*(deep learning[Title/Abstract]) OR*
*(neural networks[Title/Abstract]) OR*
*(algorithm*[Title/Abstract]) OR*
*(data-driven[Title/Abstract]) OR*
*(automated fitting[Title/Abstract]) OR*
*(closed-loop[Title/Abstract]) OR*
*(predictive modeling[Title/Abstract]) OR*
*(computational modeling[Title/Abstract]))*

**Box S2.** The composite key used in the dedicated searches.

*((cochlear implant[Title/Abstract]) OR*
*(cochlear implantation[Title/Abstract]) OR*
*(cochlear implant neural stimulation[Title/Abstract]) OR*
*(cochlear implant signal processing[Title/Abstract]) OR*
*(cochlear implant neural interface[Title/Abstract]) OR*
*(cochlear implant outcomes[Title/Abstract]) OR*
*(cochlear implant auditory pathway plasticity[Title/Abstract]) OR*
*(auditory brainstem implant[Title/Abstract]) OR*
*(ABI implantation[Title/Abstract]) OR*
*(auditory brainstem implant outcomes[Title/Abstract]) OR*
*(auditory brainstem implant neural stimulation[Title/Abstract]) OR*
*(auditory brainstem prosthesis[Title/Abstract]) OR*

| | |
|---|---|
| | *(brainstem auditory prosthesis[Title/Abstract]) OR*<br>*(advanced auditory neuroprosthesis[Title/Abstract]) OR*<br>*(auditory neuroprosthetic devices[Title/Abstract]) OR*<br>*(neural auditory prosthesis[Title/Abstract]) OR*<br>*(implantable auditory neuroprosthesis[Title/Abstract]) OR*<br>*(neuroprosthetic auditory stimulation[Title/Abstract]) OR*<br>*(next generation auditory neuroprostheses[Title/Abstract]) OR*<br>*(hybrid cochlear implant[Title/Abstract]) OR*<br>*(electro-acoustic stimulation cochlear implant[Title/Abstract]) OR*<br>*(combined acoustic and electric hearing[Title/Abstract]) OR*<br>*(hybrid auditory prosthesis[Title/Abstract]))*<br>AND<br>*((artificial intelligence[Title/Abstract]) OR*<br>*(machine learning[Title/Abstract]) OR*<br>*(deep learning[Title/Abstract]) OR*<br>*(neural networks[Title/Abstract]) OR*<br>*(algorithm*[Title/Abstract]) OR*<br>*(data-driven[Title/Abstract]) OR*<br>*(automated fitting[Title/Abstract]) OR*<br>*(closed-loop[Title/Abstract]) OR*<br>*(predictive modeling[Title/Abstract]) OR*<br>*(computational modeling[Title/Abstract]))*<br>----------------------------------------<br>*((cochlear implant[Title/Abstract]) OR*<br>*(cochlear implantation[Title/Abstract]) OR*<br>*(cochlear implant neural stimulation[Title/Abstract]) OR*<br>*(cochlear implant signal processing[Title/Abstract]) OR*<br>*(cochlear implant neural interface[Title/Abstract]) OR*<br>*(cochlear implant outcomes[Title/Abstract]) OR*<br>*(cochlear implant auditory pathway plasticity[Title/Abstract]) OR*<br>*(auditory brainstem implant[Title/Abstract]) OR*<br>*(ABI implantation[Title/Abstract]) OR*<br>*(auditory brainstem implant outcomes[Title/Abstract]) OR*<br>*(auditory brainstem implant neural stimulation[Title/Abstract]) OR*<br>*(auditory brainstem prosthesis[Title/Abstract]) OR*<br>*(brainstem auditory prosthesis[Title/Abstract]) OR*<br>*(advanced auditory neuroprosthesis[Title/Abstract]) OR*<br>*(auditory neuroprosthetic devices[Title/Abstract]) OR*<br>*(neural auditory prosthesis[Title/Abstract]) OR*<br>*(implantable auditory neuroprosthesis[Title/Abstract]) OR*<br>*(neuroprosthetic auditory stimulation[Title/Abstract]) OR*<br>*(next generation auditory neuroprostheses[Title/Abstract]) OR*<br>*(hybrid cochlear implant[Title/Abstract]) OR*<br>*(electro-acoustic stimulation cochlear implant[Title/Abstract]) OR*<br>*(combined acoustic and electric hearing[Title/Abstract]) OR*<br>*(hybrid auditory prosthesis[Title/Abstract]))* |
